# Combination of GLP‐1 receptor agonists and behavioural treatment in type 2 diabetes elicits synergistic effects on body weight: A retrospective cohort study

**DOI:** 10.1002/edm2.82

**Published:** 2019-07-26

**Authors:** Maria Letizia Petroni, Luca Montesi, Santo Colosimo, Maria Turchese Caletti, Arianna Mazzotti, Giulio Marchesini

**Affiliations:** ^1^ Department of Medical and Surgical Sciences‐DIMEC “Alma Mater” University Bologna Italy; ^2^ Department of Eating and Weight Disorders Villa Garda Hospital Garda Italy; ^3^ AUSL Diabetes Unit Romagna Ravenna Italy

**Keywords:** GLP‐1 receptor agonists, lifestyle, metabolic control, type 2 diabetes, weight loss

## Abstract

**Aims:**

Intensification of type 2 diabetes (T2DM) treatment with GLP‐1 receptor agonists (GLP‐1RAs) promotes weight loss. We aimed to determine the synergistic effect of behavioural programmes on body weight on top of GLP‐1RA treatment.

**Materials and methods:**

We retrospectively analysed the time course of 328 individuals with T2DM starting GLP‐1RA treatment because of insufficient metabolic control. In 29, a structured programme of elementary nutritional counselling was also implemented (elementary nutritional education [ENE]‐5 group sessions), whereas 53 entered a programme of cognitive‐behavioural treatment (CBT‐12 group sessions). Both programmes were completed within 6 months of switching to GLP‐1RAs. Data of body weight and metabolic control were followed up to 2 years as part of regular follow‐up. Weight loss targets (≥10% and ≥5%) and metabolic target (HbA1c < 7%) were analysed by Cox regression model in comparison with standard care (SC, N = 244).

**Results:**

Body weight remarkably decreased following both behavioural programmes, with significant differences compared with SC at 2 years (CBT, 8.5 ± 5.9% vs 6.3 ± 6.9 in ENE and only 3.1 ± 5.7 in SC; *P* < 0.001 and *P* = 0.045 vs CBT and ENE, respectively). The 10% weight loss was achieved and maintained in approximately 30% of cases during follow‐up, and an additional 35% of cases lost between 5% and 10%. Data were consistent between behavioural programmes, after adjustment for confounders, including initial body weight (logreg Mantel‐Cox: ENE vs SC, *P* < 0.01; CBT vs SC, *P* < 0.001). No differences in metabolic control were detected between groups.

**Conclusions:**

Initiation of GLP‐1RA treatment provides an opportunity for addressing patients' needs of weight control. By producing initial weight loss, patients' motivation and self‐efficacy are expected to increase and adherence to long‐term lifestyle changes might be more easily attained.

## 1 INTRODUCTION

The treatment of type 2 diabetes mellitus (T2DM) is facing totally new paradigms compared with the standards of care published only a few years ago.^1^ While metformin is maintained as first‐line therapy, second line has moved from basal insulin or insulin‐secreting agents (sulphonylureas and glinides) to the three classes of dipeptidyl‐peptidase‐4 inhibitors (DPP4‐Is), sodium‐glucose cotransporter‐2 inhibitors (SGLT2‐Is) and glucagon‐like peptide‐1 receptor agonists (GLP‐1RAs).^2^ The new treatments have several advantages compared with the old classes; in particular, they do not increase the risk of hypoglycaemia,^3^ and, limited to SGLT2‐Is and GLP‐1RAs, reduce cardiovascular risk and body weight. Cardioprotection has been demonstrated both in prospective trials^4^, ^5^ and in real‐world observational studies,^6^, ^7^, ^8^ and makes SGLT2‐Is and GLP‐1RAs. The loss of body weight is particularly large during GLP‐1RA treatment,^9^ and these drugs are the treatment of choice in the presence of obesity.^10^ A drug of the class has been approved for treatment in obesity independently of diabetes (liraglutide)^11^ and a novel drug (semaglutide) might be a likely candidate too, considering the impressive results on body weight.^12^

The treatment of obesity remains an area of intense research, but very few drugs are approved by international agencies.^13^ Weight loss remains a pivotal issue for an effective control of T2DM,^14^ but the results achieved by behavioural treatment, both in the presence and in the absence of diabetes, are generally difficult to maintain,^15^ unless patients are engaged into intense programmes aimed at calorie restriction and physical activity.^16^ Long‐term results are driven by motivation, a construct which is maintained by self‐efficacy, that is the evidence that some results may be effectively achieved, and any amount of initial weight loss may strengthen adherence to lifestyle changes.^17^ Thus, the beneficial effects of novel glucose‐lowering drugs on body weight might start a virtuous circle, comparable to the synergistic effect reported by Wadden et al^18^ for sibutramine, when combined with cognitive‐behavioural therapy (CBT) in the treatment of obesity.

In our unit, two graded programmes of lifestyle changes are operative in patients with obesity and/or T2DM.^19^ These programmes are routinely offered to patients with T2DM, either at diagnosis or at times of treatment intensification for insufficient metabolic control. We retrospectively analysed the effects of GLP‐1RAs on body weight and metabolic control in T2DM subjects, according to their participation in programmes aimed at lifestyle changes at times of treatment switch. The underlying hypothesis was that GLP‐1RA treatment might produce a much larger improvement whenever associated with behavioural support.

## 2 MATERIAL AND METHODS

### 2.1 Patients

We retrospectively analysed all T2DM cases who were prescribed a GLP‐1RA agonist in our outpatient unit since its initial approval in the Italian market (February 2008). According to initial rules dictated by the Italian Medicines Agency (AIFA), they had to fulfil definite criteria, to undergo an online monitoring system (closed on August 2010) and to adhere to follow‐up visits, initially planned after 3‐4 months and after 6‐8 months, and every 6 months thereafter. After exclusion of cases who moved from our unit to other diabetes centres (N = 23), cases who stopped treatment for adverse events (N = 12), cases who had started treatment by <6 months (N = 48) and cases who initiated insulin treatment during follow‐up—to avoid the counteracting effect on insulin on body weight (N = 6)—we could retrieve 328 cases; in this cohort, 1‐year and 2‐year follow‐up data were available for 264 and 162 cases, respectively. Their baseline values are reported in Table 1. According to our procedures, following motivational interviewing, all patients first seen inside the centre are invited to take part in structured behavioural programmes, modulated according to the severity of their weight excess and unhealthy eating pattern (see below).^19^ At any follow‐up visit, patients receive motivational reinforcement on lifestyle changes and adherence to healthy diet and habitual physical activity, and the proposal to enter a structured programme is repeated in the presence of insufficient metabolic control. The final goal of behavioural treatment is weight loss, and two different targets were considered: weight loss ≥5% and ≥10% initial body weight.

**Table 1:** Baseline characteristics of the population with type 2 diabetes at first prescription of a GLP‐1RA, grouped according to treatment programmes (means ± SD, prevalence [95% confidence interval] or median [interquartile range])

	GLP‐1RA + SC (N = 244)	GLP‐1RA + BT (N = 82)	*P* value SC vs BT	GLP‐1RA + ENE (N = 29)	GLP‐1RA + CBT (N = 53)	*P* value ENE vs CBT
Age (y)	62.2 ± 9.8	57.0 ± 10.6	<0.001	59.2 ± 10.9	55.8 ± 10.4*	0.166
Male sex (% [95% confidence interval])	59.8 [53.4‐65.6]	51.2 [40.0‐61.2]	0.197	48.3 [29.9‐64.0]	52.8 [38.8‐64.7]	0.818
Diabetes duration (y)	8.9 ± 5.3	7.9 ± 6.7	0.219	9.2 ± 5.4	7.1 ± 7.3	0.267
Weight (kg)	105.5 ± 19.2	114.0 ± 25.9	0.002	100.8^a^ ± 17.6	121.3 ± 27.0*	0.001
Height (cm)	168.1 ± 10.3	168.6 ± 9.7	0.680	167.8 ± 9.0	169.1 ± 10.1	0.568
BMI (kg/m^2^)	37.4 ± 6.4	39.9 ± 6.6	0.003	35.9^a^ ± 6.2	42.0* ± 5.8	<0.001
Overweight/Obesity I/Obesity II/Obesity III (%)	6/35/32/27	3/26/26/45	0.018	10/48/24/17	0/13/26/60	<0.001
Waist circumference (cm)	115.9 ± 11.2	120.8 ± 14.7	0.076	111.8 ± 11.0	125.8 ± 14.4*	0.020
Blood glucose (mg/dL)	166.9 ± 50.4	159.9 ± 45.8	0.406	164.5 ± 42.7	157.8 ± 47.2	0.644
HbA1c (%)	8.06 ± 1.18	8.17 ± 1.37	0.556	8.2 ± 1.22	8.15 ± 1.45	0.878
DM at GLP‐1RA switch (SG/DPP4‐Is/PIO/INS) (%)	44/10/9/19	42/6/6/18	0.967	45/5/19/9	41/5/2/23	0.002
Total cholesterol (mg/dL)	181.8 ± 41.1	191.0 ± 41.9	0.135	199.1 ± 47.3	187.6 ± 39.6	0.335
Triglycerides (mg/dL)	197.3 ± 151.4	221.5 ± 150.0	0.292	191.5 ± 81.4	234.2 ± 170.4	0.329
HDL (mg/dL)	44.7 ± 11.0	44.1 ± 10.9	0.705	50.0 ± 13.9	41.9 ± 8.9	0.013
LDL (mg/dL)	114.6 ± 42.6	106.6 ± 34.0	0.407	114.0 ± 34.8	103.6 ± 33.6	0.319
Creatinine (mg/dL)	0.84 ± 0.21	0.81 ± 0.17	0.282	0.84 ± 0.13	0.78 ± 0.19	0.250
Microalbuminuria (mg/dL, median [IQR])	11.8 [33.0]	9.0 [19.2]	0.334	11.2 [19.3]	9.0 [16.0]	0.983
Systolic pressure (mmHg)	133.7 ± 14.9	135.9 ± 14.9	0.285	135.9 ± 17.3	135.8 ± 13.4	0.979
Diastolic pressure (mmHg)	82.0 ± 8.5	85.1 ± 8.8	0.009	85.9* ± 10.0	84.6 ± 8.1	0.554
Type of GLP‐1RA (% [95% confidence interval])						
Exenatide BID	11.5 [7.9‐15.9]	8.5 [3.8‐15.8]	0.352	13.8 [4.5‐28.5]	5.7 [1.5‐14.1]	0.131
Liraglutide	54.9 [48.4‐60.8]	63.4 [52.0‐72.5]		48.3 [29.9‐64.0]	71.7 [57.4‐81.3]	
Exenatide ER	14.3 [10.3‐19.1]	15.9 [9.0‐24.6]		17.2 [6.5‐32.6]	15.1 [7.2‐26.0]	
Dulaglutide	19.3 [14.6‐24.5]	12.2 [6.3‐20.3]		20.7 [8.7‐36.5]	7.5 [2.4‐16.6]	

Note

Metformin was background treatment in nearly all cases, and its use was evenly distributed across groups. Other treatments were not mutually exclusive.

Abbreviations: BT, behavioural therapy; CBT, cognitive‐behavioural therapy; ENE, elementary nutritional education; IQR, interquartile range; SC, standard care.

a SG, sulphonylureas and glinides; DPP4‐Is, dipeptidyl‐peptidase‐4 inhibitors; PIO, pioglitazone; INS, basal insulin. Metformin was background treatment in nearly all cases, and its use was evenly distributed across groups. Other treatments were not mutually exclusive.

* *P* < 0.05 vs GLP‐1RA + SC.

The analysis of the effects of GLP‐1RA treatment on body weight and metabolic control configures as an internal audit, and does not require informed consent by patients. The analysis was notified to the ethical committee of the Azienda Ospedaliero‐Universitaria, Bologna (study COMBINATION‐R, Prot. N. 182.2017.O.OssN).

### 2.2 Behavioural programmes

In addition to nutritional counselling by the physicians or dieticians (when first seen in the centre), two structured behavioural programmes are operative in our centre: (a) elementary nutritional education (ENE) and (b) cognitive‐behavioural therapy (CBT). The first programme is carried out during five weekly 120‐minutes sessions, in groups of 20‐25 persons. Lessons are aimed at lifestyle changes and cover several aspects of nutrition and physical activity with the support of a residential manual: (a) energy balance, nutrients and weight monitoring; (b) alimentary pyramid and size of portions; (c) food shopping and food labels; and (d) physical activity, when and how much. The last session is chaired by a psychologist and deals with relapse. The second, more intensive programme is carried out in 12 weekly sessions (120 minutes each), based on the LEARN program for weight control,^20^ also supported by a residential manual.^21^ Group sessions (12‐15 subjects) extend the information given in ENE, but subjects are also instructed on the principles of calorie counting, monitoring their daily food intake (eating dairy), on behavioural strategies for stimulus control, and towards a pattern of regular eating. Two sessions are on identifying and coping with dysfunctional cognition with the support of a psychologist. Cognitive strategies focus on (a) identifying high‐risk situations responsible for binge eating and regaining control towards a pattern of regular eating; (b) learning problem‐solving skills and identifying and coping with dysfunctional cognition; and (c) maintaining improvement and preventing relapse.

The reason(s) for programme selection depend both on physicians' evaluation (patients' age, education, degree of obesity, previous compliance to treatments, cognitive status) and on patients' preferences (refusal of more intense interventions because of time or job constraints, living far from the centre, etc). Patients who do not participate in behavioural programmes are invited to adhere to a personalized diet and are also encouraged to increase their physical activity to a target level of at least 30 minutes five times a week (standard care—SC).

### 2.3 Measures

At baseline and at any control visits, body weight and height, waist circumference and blood pressure were available,^22^ as well as blood glucose, lipid profile (total cholesterol, HDL cholesterol, triglycerides), HbA1c (HPLC method) and renal function (creatinine, glomerular filtration rate [eGFR] and microalbuminuria). A programme for the standardization of biochemical assays has been active among Bologna laboratories since 2008, and has later turned into a Laboratorio Unico Metropolitano, serving the whole area of Bologna.

### 2.4 Statistical analysis

All data were initially anonymised, implemented on an Excel database and analysed using StatView 5.0™ statistical package (SAS Institute Inc) and SPSS v.20 (IBM Company). A descriptive analysis was initially carried out by computing mean and standard deviation, or median and interquartile range for non‐normally distributed data, as well as prevalence and 95% confidence interval for nominal characteristics. All values were also calculated for the different cohorts grouped according to participation into the different behavioural programmes and tested for baseline differences by ANOVA. Time‐to‐target reach according to treatment programmes was compared between study groups with the use of a Wald test for the estimated hazard ratio from a Cox proportional‐hazards model with a single covariate for the study group. The Wald test was chosen to provide consistency between reported *P* values and 95% confidence intervals. Rates of target reach were analysed by the Kaplan‐Meier method. Multivariate Cox proportional‐hazards models on the basis of baseline data were also used to evaluate time to target while adjusting for potentially significant risk factors. Finally, logistic regression analyses were used to identify the determinants of target reach at 1 and 2 years of continuous treatment, after adjusting for confounders (age, sex, BMI, diabetes duration, metabolic control, baseline drug treatment), considering that in a few cases weight regain occurred. *P* values <0.05 were considered statistically significant.

## 3 RESULTS

### 3.1 Baseline characteristics (Table 1)

All cases switched to GLP‐1RAs because of insufficient metabolic control attained by metformin with/without other oral glucose‐lowering agents (sulphonylureas or glinides, pioglitazone or, more recently, DPP‐4 inhibitors) and/or basal insulin. Metformin use, considered background treatment, was evenly distributed across groups. Whereas DPP‐4 inhibitors were always stopped, sulphonylureas, glinides and pioglitazone were frequently maintained and tapered down during follow‐up. As expected, patients with T2DM entering the behavioural programme were characterized on average by younger age and more severe obesity compared with patients followed by SC after switching to GLP‐1RA. These differences were particularly present in the cohort of cases who agreed to participate in the more intensive CBT protocol, who were also characterized by different preswitching drug use when compared with individuals entering the ENE protocol. No systematic differences were present at baseline in metabolic control (HbA1c levels), lipid profile and renal function, including the presence of albuminuria.

Liraglutide was the most commonly used drugs (over 50% of cases). It was used at a final dose of 1.2 mg/d in 52 cases (SC, 35; BT, 17; *P* = ns) and at the maximum dose of 1.8 mg/d in 120 cases (SC, 87; BT, 33; *P* = ns). Other GLP‐1RAs were always used at the standard dose of 10 mcg bid (exenatide), 2 mg/wk (exenatide ER) and 1.5 mg/wk (dulaglutide).

### 3.2 Effects of treatment on body weight and target reach

**Figure 1:**
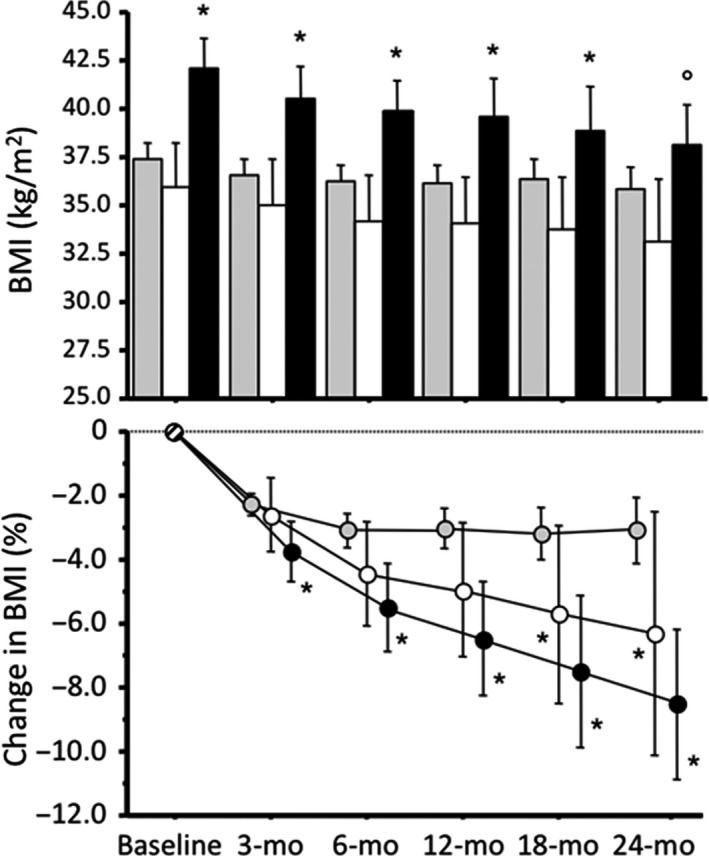
Body mass index (upper panel) and percentage decrease in body mass index (lower panel) at baseline and in the course of follow‐up in subjects treated by GLP‐1RA, according to participation in the different behavioural programmes. Data are presented as means ± 95% confidence intervals. Note that grey bars and circles correspond to cases treated by standard medical care (SC), white bars and circles represent cases treated by the ENE programme, and black columns and circles are cases who attended the CBT programme. BMI decreased systematically at any time point compared with the corresponding value at baseline. *P < 0.05 vs SC; °*P* < 0.05 vs ENE

GLP‐1RA treatment was associated with a systematic reduction in body weight in all cohorts (Figure 1). Body weight loss was much larger in subjects entering the superimposed behavioural treatment, and particularly in the CBT cohort, where it was on average 6.5 ± 5.8% in subjects reaching the 1‐year observation period and 8.5 ± 5.9% after 2 years (vs 6.3 ± 6.9% in ENE and only 3.1 ± 5.7% in SC after 2 years; *P* < 0.001 and *P* = 0.045 vs CBT and ENE, respectively).

**Figure 2:**
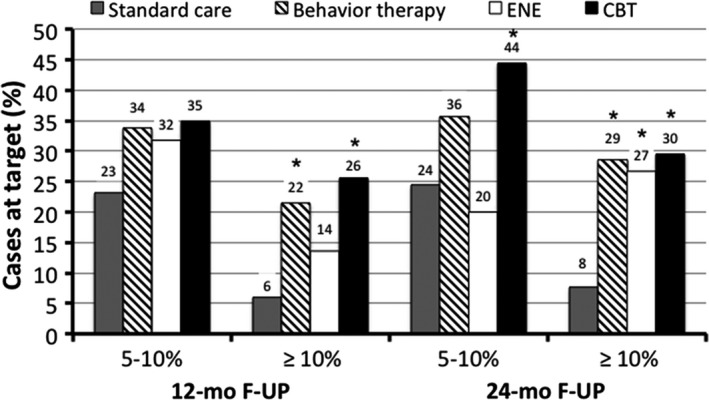
Weight loss target reach in subjects treated according to the different programmes. Behavioural treatment is the sum of cases treated according to the two different lifestyle programmes, characterized by different intensity. *P < 0.05 vs standard care

At 1‐year follow‐up, the 10% weight loss target was achieved in only 6% of cases in SC, vs 14% and 26% in the ENE and CBT groups, respectively (*P* < 0.001); after 2 years, only 8% of cases treated by SC achieved the 10% weight loss, vs 27% in ENE and 30% in CBT (*P* < 0.05 for both) (Figure 2).

**Figure 3:**
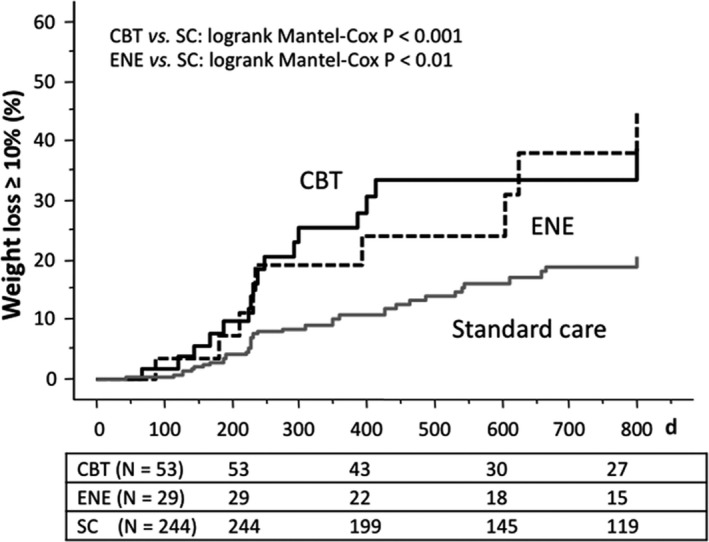
Ten per cent weight loss target reach during GLP‐1RA treatment, according to superimposed behavioural programme. Abbreviations: CBT, cognitive‐behavioural treatment; ENE, elementary nutritional education; SC, standard care

In a multivariate Cox proportional‐hazards model, the probability to achieve the 5% weight loss was significantly increased neither by adding behavioural treatment to the initiation of GLP‐1RA treatment, nor by any of the two treatment programmes (Table 2). On the contrary, any behavioural treatment more than doubled the probability to achieve 10% weight loss (Table 2, Figure 3), and the results were maintained after adjustment for principal confounders, namely age, gender, initial HbA1c and, particularly, BMI. No differences were demonstrated in relation to the type of GLP‐1RA (short‐acting, exenatide bid; once‐daily liraglutide; once‐weekly exenatide ER or dulaglutide).

**Table 2:** Multivariate Cox proportional‐hazards model of time‐to‐target reach (weight loss ≥5% and ≥10% of initial body weight) according to treatment programmes

Treatment programme	≥5% weight loss		≥10% weight loss	
	Coef/SE	Exp(Coef) [95% CI]	Coef/SE	Exp(Coef) [95% CI]
GLP‐1RA + SC (reference)	–	–	–	–
GLP‐1RA + BT	0.284	1.06 [0.88‐1.55]	3.373	**2.43 [1.45‐4.08]**
GLP‐1RA + BT (adjusted)^a^	0.075	1.02 [0.66‐1.56]	2.926	**2.26 [1.31‐3.92]**
GLP‐1RA + ENE	1.000	1.31 [0.77‐2.24]	2.294	**2.36 [1.13‐4.93]**
GLP‐1RA + ENE (adjusted)^a^	0.728	1.24 [0.70‐2.20]	2.265	**2.43 [1.13‐5.26]**
GLP‐1RA + CBT	−0.392	0.90 [0.55‐1.50]	2.975	**2.47 [1.36‐4.47]**
GLP‐1RA + CBT (adjusted)^a^	−0.763	0.81 [0.46‐1.40]	2.237	**2.10 [1.10‐4.03]**

Note

Standard care was used as control treatment. Statistically significant values are presented in bold characters. No significant differences were measured in comparison with GLP‐1RA + ENE vs GLP‐1RA + CBT.

Abbreviations: BT, behavioural treatment; CBT, cognitive‐behavioural treatment; ENE, elementary nutritional education; SC, standard care.

a Adjusted for age, gender and initial BMI.

A sensitivity analysis was limited to subjects with obesity grades II‐III. In this subset of patients, no differences in BMI were observed either in relation to superimposed behavioural programmes (SC, 41.4 ± 5.6 kg/m^2^, N = 137; behavioural treatment, 43.0 ± 5.2 kg/m^2^, N = 57; *P* > 0.05), or in relation to ENE vs CBT (ENE, 42.1 ± 5.5 kg/m^2^, N = 11; CBT, 43.3 ± 5.2 kg/m^2^, N = 46; *P* > 0.05). With the limits of the lower sample size, BT was again associated with a higher probability to achieve the 10% weight loss at 2 years (odds ratio [OR], 2.46; 95% confidence interval [CI], 1.30‐2.67), and this was maintained both in subjects treated by the CBT programme (OR, 2.35; 95% CI, 1.18‐4.65) and in those treated by ENE (OR, 2.99; 95% CI, 1.02‐8.78).

In a few cases, weight loss fluctuated during follow‐up; in particular, five patients (two in the CBT group, one in ENE and two in SC) who reached the 10% weight loss target at 1 year were no longer at target after 2 years. Nonetheless, the two lifestyle programmes were the only variables associated with 10% weight loss at 2 years (ENE: odds ratio, 5.87 [95% confidence interval, 1.15‐29.92]; CBT: odds ratio, 8.97 [2.17‐37.04]; *P* < 0.001 for both), after adjustment for age, gender, BMI and HbA1c at baseline.

### 3.3 Effects on metabolic control

HbA1c decreased rapidly in response to GLP‐1RA treatment, on average by 0.98 ± 1.22% and 0.94 ± 1.27% after 1 and 2 years, respectively, without differences between treatment programmes. After 1 year, the standard HbA1c target of 7% was achieved in 36% of cases treated by GLP‐1RA + SC, in 38% following GLP‐1RA + ENE and 40% following GLP‐1RA + CBT (not different) and no differences were also observed after 2 years (on average, 53%).

Changes in HbA1c were also driven by the different use of other glucose‐lowering drugs. The use of sulphonylureas/repaglinide declined remarkably in the three cohorts, as was the case of basal insulin and pioglitazone, with minor differences between groups (Table 3).

**Table 3:** Treatment with glucose‐lowering drug before switching to GLP‐1RAs (Pre) and in addition to GLP‐1RAs during the observation study (basal, 1 y, 2 y)

Cohort	Sulphonylureas/repaglinide (%)				Pioglitazone (%)				Basal insulin (%)		DPP‐4Is (%)
Time^a^	Pre	Basal	1‐y	2‐y	Pre	Basal	1‐y	2‐y	Pre	Basal^b^	Pre^b^
GLP‐1RA + SC	43.7	31.7	29.5	25.0	9.3	5.9	7.7	7.3	19.1	14.2	9.9
GLP‐1RA + ENE	45.5	22.7	18.2	4.8	14.3	13.7	13.7	9.5	9.1	9.1	4.5
GLP‐1RA + CBT	40.9	25.0	22.7	29.3	2.3	2.3	4.7	2.5	22.7	11.6	4.5
Total	43.4	29.8	27.3	23.9^c^	8.9^c^	6.3	7.7	7.3	18.9	13.3	8.4

Abbreviations: CBT, cognitive‐behavioural treatment; ENE, elementary nutritional education.

a A number of cases are as follows: GLP‐1RA + SC, 244 (Pre and basal), 199 (1 y) and 119 (2 y); GLP‐1RA + ENE, 29, 22 and 15; GLP‐1RA + CBT, 53, 43 and 27, respectively.

b Basal insulin was maintained in association with GLP‐1RAs in all cases, although at variable doses; DPP4‐Is were always stopped at time of switch to GLP‐1RA treatment; metformin was used in nearly all cases (excluding intolerant patients) at standard doses of 2000‐2550 mg/d.

c The analysis indicates heterogeneity of use among groups (Chi^2^, *P* value <0.05).

The effects on metabolic control are more clearly demonstrated in the cohort observed during the whole period of study (Table S1). In these patients, a systematic decline in HbA1c was observed, without differences in relation to behavioural treatment, accompanied by minor changes in lipid levels. Systolic blood pressure did not vary, whereas diastolic pressure decreased significantly in all groups.

## 4 DISCUSSION

The report shows how much a behavioural programme may help increase weight loss in association with GLP‐1RA treatment in motivated patients. In T2DM, weight loss is highly desirable; when combined with behavioural programmes, GLP‐1RA‐associated weight loss continues to increase during the 2‐year observation period, up to values rarely achieved by individual treatments. Combination treatment is expected to reduce the burden of disease, the progression of comorbidities and eventually mortality.^23^

The beneficial effects of GLP‐1RA treatment on body weight have been extensively investigated in registration trials and in the real world. In a systematic review of the literature up to May 2011 of 18 clinical trials in T2DM people with BMI ≥ 25 kg/m^2^, Vilsboll et al^24^ reported a weighted mean difference in body weight of −2.8 kg in comparison with control treatment, without differences in relation to initial BMI or trial duration. These results have been confirmed in 2015 by a systematic review and mixed treatment comparison meta‐analysis, where different GLP‐1RA therapies (exenatide 20 μg daily, exenatide 2 mg/wk, liraglutide 1.2 mg daily and liraglutide 1.8 mg daily) were compared with other glucose‐lowering drugs.^25^ Real‐world Italian data confirmed an average weight loss of 3.5% with continuous long‐term use of exenatide bid treatment,^26^ but larger effects of on body weight were reported with liraglutide, supporting the use of the drug for the treatment of obesity.^27^ The advent of weekly injectable GLP‐1RA has opened a new era for the treatment of T2DM: both exenatide ER and dulaglutide are now available, with effects on body weight not systematically different from liraglutide (depending on treatment dosage),^28^ whereas the effects of semaglutide might be even larger.^29^ Notably, GLP‐1RA has been used as add‐on to behavioural treatment of obesity: in the Maintenance SCALE randomized study, liraglutide was effective in favouring weight loss maintenance and additional weight loss when superimposed to a low‐calorie weight loss programme.^30^

The present study was addressed to test the opposite mechanism, that is, the effectiveness of weight loss programmes to exploit the effects of GLP‐1RA on body weight. Behaviour‐induced weight loss strongly depends on adherence to and persistence in healthy, calorie‐restricted diet and physical activity,^31^ in turn favoured by self‐efficacy.^32^ The initial weight loss induced by GLP‐1RA treatment is likely to strengthen self‐efficacy, enhancing the effects of behavioural programmes. In keeping with this hypothesis, body weight continued to decline on average in patients participating in behavioural programmes, and the percentage of cases reaching the challenging 10% weight loss target increased progressively in the 2‐year observation period to 29%, with an additional 36% achieving the 5% target. Notably, the probability to reach the weight loss target of 5% was rather high also in subjects on SC (24%), which explain the lack of statistical difference when compared to BT. Per protocol, two behavioural programmes of different intensity are offered to patients with T2DM in poor metabolic control at our department; they were reported to facilitate metabolic control as well as weight loss and to retard insulin treatment.^19^ A reanalysis of those historical data showed that ENE produced a weight loss of 4% at 1 year (vs 14% in the present series, *P* = 0.051) and 9% at 2 years (vs 27%, *P* < 0.05), whereas CBT resulted in a weight loss of 18% and 20%, respectively.^19^ These figures are remarkably lower than the percentage of cases at target by the simultaneous GLP‐1RA initiation, in keeping with an additive effect.

The study has limits, which should be discussed. First, the report is an audit of patients receiving treatment in a single department, not a randomized controlled study, and is prone to several biases in spite of multiple adjustments. Second, the *per‐protocol* analysis was based on patients' adherence to the behavioural programmes, proposed to patients on the basis of their poor metabolic control and the degree of obesity. These cohorts might include subjects more motivated to weight loss, thus biasing the comparison with SC. However, the analysis of the historical cohort indicates that also in comparison with equally motivated patients GLP‐1RA treatment may significantly enhance the beneficial effects on body weight. Several studies identified early weight loss as predictor of long‐term outcome^33^, ^34^; accordingly, GLP‐1RA‐associated pleiotropic effects might drive and maintain long‐term lifestyle changes and weight loss. Notably, this was mainly observed in patients treated by the less intensive programme, including subjects with less severe obesity grades, who are less concerned by excess body weight. Third, we could not detect any systematic effect of the different treatment programmes on metabolic control, in spite of the different weight loss. At time of switch to GLP‐1RA treatment, large differences in HbA1c were present among individual patients and across groups, and the final target was set at values different in relation to social and clinical parameters, as suggested by most recent Italian and international guidelines.^35^, ^36^ Accordingly, the use of other glucose‐lowering drugs was also modified, possibly blurring the final effects in the retrospective analysis. Finally, we did not systematically measure food intake and physical activity to associate weight loss with lifestyle changes.

In conclusion, the initiation of GLP‐1RA treatment might be a pivotal starting point to accompany motivated subjects with T2DM towards lifestyle changes. Both gastrointestinal symptoms and the possible effects of these drugs on the central mechanism(s) of satiety might start and enhance a virtuous circle leading to large and persistent weight loss when accompanied by behavioural programmes. A randomized controlled trial has been recently set up to support these retrospective data.

## CONFLICT OF INTERESTS

All authors declare no conflict of interest in relation to the material presented in this study.

## AUTHOR CONTRIBUTIONS

GM, LM and MLP conceived the study; LM, SC, MTC, AM collected the data; MLP, SC and GM were involved in statistical analyses and drafted the manuscript; all authors contributed substantially to its revision and agreed to be accountable for all the aspects of the work; and GM takes responsibility for the paper as a whole.

## ETHICAL APPROVAL

The anonymous analysis represents an internal audit, and does not require signed consent by patients. The study plan was notified to the ethical committee of the Azienda Ospedaliero‐Universitaria, Bologna (study COMBINATION‐R, Prot. N. 182.2017.O.OssN).

## Data Availability

The whole set of data is available upon request.
