# Supplementary material for: Combination of GLP‐1 receptor agonists and behavioural treatment in type 2 diabetes elicits synergistic effects on body weight: A retrospective cohort study
Source: Endocrinol Diabetes Metab. 2019 Jul 26;2(4):e00082. doi: 10.1002/edm2.82 (PMC6775466; doi:10.1002/edm2.82)
Supplement: Supplementary file 1 [file EDM2-2-e00082-s001.docx]

**Supplementary Table**

Time-course of metabolic parameters and arterial pressure in the selected cohort reaching the 2-year follow-up.

| Variable | GLP-1RA+SC (N=112) | GLP-1RA+ENE (N=10) | GLP-1RA+CBT (N=24) | ANOVA |
| --- | --- | --- | --- | --- |
| Glycosylated hemoglobin (%) | |  |  |  |
| Baseline | 8.00 ± 1.08 | 8.28 ± 0.69 | 8.64 ± 1.33 | Time: P<0.001  Time x treatment: P=0.560 |
| 1-year | 7.13 ± 0.90 | 6.88 ± 0.83 | 7.21 ± 1.06 |  |
| 2-year | 7.05 ± 0.95 | 7.22 ± 1.23 | 7.42 ± 1.44 |  |
| Total cholesterol (mg/dL) |  |  |  |  |
| Baseline | 179.0 ± 35.7 | 167.8 ± 40.0 | 204.2 ± 39.7 | Time: P=0.210  Time x treatment: P=0.010 |
| 1-year | 169.9 ± 35.8 | 182.1 ± 27.5 | 185.0 ± 35.7 |  |
| 2-year | 172.1 ± 40.2 | 174.6 ± 28.7 | 172.9 ± 32.4 |  |
| HDL-cholesterol (mg/dL) |  |  |  |  |
| Baseline | 44.8 ± 11.9 | 47.8 ± 13.1 | 43.2 ± 9.1 | Time: P=0.027  Time x treatment: P=0.286 |
| 1-year | 45.9 ± 11.5 | 46.7 ± 9.9 | 42.0 ± 8.3 |  |
| 2-year | 45.7 ± 11.2 | 45.6 ± 6.9 | 43.8 ± 8.6 |  |
| Triglycerides (mg/dL) |  |  |  |  |
| Baseline | 185.6 ± 114.6 | 160.0 ± 60.1 | 245.4 ± 185.9 | Time: P=0.073  Time x treatment: P=0.939 |
| 1-year | 155.5 ± 76.0 | 172.9 ± 106.6 | 178.1 ± 89.8 |  |
| 2-year | 155.5 ± 67.6 | 209.3 ± 203.5 | 151.1 ± 75.1 |  |
| Systolic pressure (mmHg) |  |  |  |  |
| Baseline | 134.4 ± 11.2 | 133.0 ± 12.0 | 138.3 ± 2.9 | Time: P=0.796  Time x treatment: P=0.286 |
| 1-year | 130.4 ± 11.1 | 134.0 ± 15.6 | 133.3 ± 5.8 |  |
| 2-year | 130.5 ± 13.1 | 131.0 ± 9.7 | 132.0 ± 8.7 |  |
| Diastolic pressure (mmHg) |  |  |  |  |
| Baseline | 83.3 ± 7.3 | 85.8 ± 5.1 | 88.3 ± 10.4 | Time: P=0.015  Time x treatment: P=0.533 |
| 1-year | 79.8 ± 8.0 | 78.0 ± 13.5 | 82.3 ± 2.5 |  |
| 2-year | 77.3 ± 7.6 | 82.0 ± 7.9 | 76.0 ± 6.0 |  |

Data at baseline and at one-year follow-up were not different from those observed in the total cohort under study,
